# The comparison of largemouth bass (*Micropterus salmoides*) fed trash fish and formula feeds: Growth, flesh quality and metabolomics

**DOI:** 10.3389/fnut.2022.966248

**Published:** 2022-09-30

**Authors:** Xiaoying Xu, Hang Yang, Zhen Xu, Xiaoqin Li, Xiangjun Leng

**Affiliations:** ^1^National Demonstration Center for Experimental Fisheries Science Education, Shanghai Ocean University, Shanghai, China; ^2^Center for Research on Environmental Ecology and Fish Nutrition (CREEFN) of the Ministry of Agriculture, Shanghai Ocean University, Shanghai, China; ^3^Shanghai Collaborative Innovation for Aquatic Animal Genetics and Breeding, Shanghai Ocean University, Shanghai, China

**Keywords:** largemouth bass, trash fish, formula feed, growth, flesh quality, metabolomics

## Abstract

This study compared the growth, flesh quality and metabolomics of largemouth bass (*Micropterus salmoides*) fed trash fish and formula feeds. Trash fish (TF), self-made feed (SF) and commercial feed (CF) were prepared with crude protein levels of 172.2 g/kg, 503.5 g/kg and 504.1 g/kg (666.2 g/kg, 547.3 g/kg and 535.1 g/kg based on dry matter), respectively. Then, the three diets were fed to largemouth bass with an initial body weight of 75.0 ± 0.1 g for 12 weeks. SF and CF groups presented significantly lower feed intake (FI), feed conversion ratio (FCR) and higher protein efficiency ratio (PER) than TF group based on dry matter basis without affecting the weight gain (*P* < 0.05). The yellowness (b*) in dorsal muscle, flesh heat-insoluble collagen and free flavor amino acids contents in SF group were significantly higher (*P* < 0.05), while drip loss were significantly lower (*P* < 0.05) than those of TF group. Compared to TF group, SF and CF groups showed significantly higher flesh polyunsaturated fatty acids (PUFAs), n-3 PUFAs and n-6 PUFAs contents, flesh hardness, shear force and muscle fiber density (*P* < 0.05), and lower flesh total free amino acids, essential amino acids, muscle fiber diameter, intestine villus height and muscular thickness (*P* < 0.05). The serum total protein, triglyceride and cholesterol levels in SF group were significantly lower than those in TF and CF groups (*P* < 0.05). In the muscle metabolomics, 177 differential metabolites were detected between SF and TF groups, which mainly enriched in pathways as biosynthesis of amino acid, histidine metabolism, glycine, serine and threonine metabolism, etc. Conclusively, feeding largemouth bass with formula feeds improved flesh fatty acid profile and flesh texture without negative effects on the growth, but the flesh free amino acids contents were lower than the fish fed trash fish.

## Introduction

Largemouth bass (*Micropterus salmoid*es) is a typical freshwater carnivorous fish with fast growth, delicious taste and high nutritional value. In recent years, the aquaculture of largemouth bass in China has developed rapidly, and the production reached 619,519 tons in 2020 (1). In the early period, largemouth bass was cultured by feeding trash fish or trash fish + formula feed. The successful preparation of formula feed makes it possible to completely replace trash fish in largemouth bass aquaculture during the whole feeding period. However, some farmers still use trash fish periodically due to the concerns about growth and flesh quality. However, feeding trash fish may carry some pathogenic bacterial and viral pathogens to induce serious diseases for farmed aquatic species (2). Long-term use of trash fish in aquaculture is more likely to worsen water quality, resulting in environmental problems as eutrophication (3). Thus the replacement of trash fish by formula feeds will be a trend in future aquaculture.

Some studies have compared the growth performance (4), flesh crude protein, amino acids and fatty acids (5), liver and intestinal tissue structure (4), intestinal digestive enzyme activity (6) and intestinal microflora (7) of largemouth bass fed trash fish and fed formula feed. The flesh quality comparisons of trash fish fed fish and formula diets fed fish have been reported in carnivorous fishes as snakehead (*Channa striata*) (8, 9), large yellow croaker (*Larimichthys crocea)* (10–12), grouper (*Epinephelus coioides*) (13), etc. Hien et al. (8) found that feeding trash fish and formula feed did not significantly affect the flesh taste and texture (hardness, chewiness) of snakehead. In large yellow croaker, formula diet fed fish showed higher flesh hardness, total hydroxyproline, flesh n-6 PUFAs, total free amino acids and flavor amino acids levels than trash fish fed fish (11). In sea bass (*Lates calcarifer*), the flesh essential fatty acids such as n-3 polyunsaturated fatty acid, DHA and EPA of fish fed with sinking diet were significantly higher than those of fish fed with trash fish, and the fish fed with commercial floating diet also showed better flesh odor, taste and hardness than those fed with trash fish (14). Li et al. (5) once reported higher flesh linoleic acid content of largemouth bass fed with formula diet than fish fed with trash fish, but the quality of flesh protein and amino acid was lower. However, the systematic comparison in flesh quality of formula diet fed largemouth bass and trash fish fed largemouth bass has not been reported, including nutritional value, flavor, taste, flesh water holding capacity, flesh texture and muscle histology, which has attracted more and more attentions from farmers and consumers.

At present, nutrition metabolomics of aquatic animal has been applied to analyze the feeding pattern (15, 16), nutritional requirements (17), feed additives (18) and feed ingredient substitution (19, 20), providing a new direction and platform for the research of aquatic animal nutrition. However, few studies were reported about the effects of dietary trash fish and formula feeds on flesh metabolomics and the relationship between flesh quality and metabolite changes. Therefore, the present study comprehensively compared the growth, flesh quality and metabolomics of largemouth bass fed trash fish and formula feeds (commercial feed and self-made feed), directing the application of artificial feed in largemouth bass aquaculture.

## Materials and methods

### Ethical statement

All experimental animal care protocols were approved by the Institutional Animal Care and Use Committee (IACUC). All procedures were strictly carried out in accordance with the Regulations of the Experimental Animal Ethics Committee of Shanghai Ocean University.

### Experimental diets and design

Three diets were designed as trash fish (TF), commercial feed (CF) and self-made feed (SF) by our lab. The trash fish was ious fish (*Hemiculter leucisculus*) purchased from a local market in Pudong (Shanghai, China) and stored at –20°C until use. The commercial feed was floating feed produced by Tongwei Co., Ltd. Self-made feed consisted of fish meal (40%), meat and bone meal (5%), soy protein concentrate (8%), corn gluten meal (8%), wheat gluten (6%), wheat flour (15%), brewer’s yeast (4%), fish oil (2%), soybean oil (2.5%), soybean lecithin (2.5%), squid paste (4%), vitamin and mineral mixtures (3%). All ingredients were ground and screened through a 60-mesh sieve, then gradually mixed and granulated into sinking pellet with diameter of 4.0 mm (Extruder LX-75, Longxiang Food Machinery Factory, Hebei, China). The pelleting temperature was 90 ± 5°C. All diets were air-dried and stored at 4°C until use. The proximate composition and amino acid composition of diets are shown in Table 1, and fatty acid composition is shown in Table 2.

**TABLE 1:** Proximate composition and amino acid composition of the experimental diets (g/kg).

	TF	CF	SF
Proximate composition*			
Crude protein	172.2 (666.2)	504.1 (535.1)	503.5 (547.3)
Crude lipid	46.8 (181.2)	104.6 (111.0)	124.3 (135.2)
Ash	39.2 (151.7)	132.9 (141.1)	94.1 (102.3)
Moisture	741.5	58.0	80.0
**Amino acid composition (dry matter basis)**			
Aspartic acid	61.6	47.7	43.7
Threonine	28.0	19.3	20.2
Serine	26.4	22.1	24.8
Glutamic acid	92.2	73.2	87.4
Glycine	44.1	37.1	24.5
Alanine	40.8	32.8	30.3
Cysteine	4.0	1.8	3.1
Valine	26.4	13.8	17.1
Methionine	15.6	9.1	11.9
Isoleucine	24.1	11.2	13.0
Leucine	50.8	37.0	42.2
Tyrosine	19.9	16.4	18.8
Phenylalanine	27.7	29.8	29.2
Lysine	54.4	41.2	30.1
Histidine	17.4	17.0	14.4
Arginine	41.7	31.5	25.5
Proline	26.8	25.8	29.6
EAAs	284.5	212.6	208.2
TAAs	602.1	466.7	465.7

*self-made feed, commercial feed were air-dried weight, trash fish was fresh weight. Data in parentheses is nutrient content based on dry matter basis.

**TABLE 2:** Fatty acids composition of the experimental diets (percentage of fatty acids, %).

	TF	CF	SF
C14: 0	2.12	1.74	1.75
C15: 0	0.80	0.25	0.21
C16: 0	12.76	12.38	11.75
C17: 0	1.08	0.28	0.25
C18: 0	3.62	3.42	3.12
C20: 0	0.22	0.26	0.22
C16: 1	8.41	3.55	3.62
C18: 1	30.79	24.16	21.76
C20: 1	1.79	2.36	1.84
C18: 2	15.66	27.61	26.26
C18: 3	4.37	3.14	3.06
C20: 2	1.09	—	—
C20: 3	2.34	0.59	2.87
C20: 4	3.40	1.13	1.28
C20: 5 (EPA)	3.46	7.32	8.90
C22: 5	2.79	1.23	1.39
C22: 6 (DHA)	5.29	10.59	11.72
SFAs	20.61	18.32	17.30
MUFAs	41.00	30.06	27.22
PUFAs	38.39	51.61	55.48
n-3 PUFAs	15.91	22.29	25.07
n-6 PUFAs	22.48	29.32	30.41

SFAs, saturated fatty acids; MUFAs, monounsaturated saturated fatty acids; PUFAs, polyunsaturated fatty acids.

### Experimental fish and feeding management

Largemouth bass were bought from Linghu Fengmin Aquafarm in Huzhou (Zhejiang, China). Before the start of the trial, the fish were stocked in pools to acclimatize to the experimental environment for 2 weeks. At the start of the feeding trial, all fish were deprived of diets for 24 h, then a total of 135 fish with an average initial weight of 75.0 ± 0.1 g were randomly distributed into 9 round polypropylene tanks (650 L, 1.0 m × 0.8 m) with three replicates (tanks) per treatment and 15 fish per tank. In the circulating system, the water was aerated with a flowing rate of 10 L/min per tank.

During the feeding period, the fish were fed manually to apparent satiation twice per day (7:30 a.m., 16:30 p.m.). In trash fish group, the frozen trash fish were thawed at room temperature and cut into suitable blocks before feeding. Uneaten feeds were collected in 30 min after feeding, then oven dried and weighed to calibrate the feed intake. For trash fish residue, both wet weight and dry weight were recorded to calculate feed conversation ratio based on fresh weight and dry matter. The feces waste was siphoned out every day, and about 1/3 of water was renewed every 3 days. The water quality was monitored every day, and the temperature, dissolved oxygen, pH, ammonia nitrogen and nitrite of water were 24–31.0°C, > 5 mg/L, 7.5–8.0, < 0.2 mg/L and < 0.1 mg/L, respectively. The feeding experiment was conducted in the indoor circulating water system at Binhai Aquaculture Station of Shanghai Ocean University (Shanghai, China) with a feeding period of 12 weeks.

### Samples collection

Prior to slaughter, all fish were deprived of diets for 24 h, then counted and weighed for each tank to calculate weight gain (WG), feed conversion ratio (FCR), survival, feed intake (FI) and protein efficiency ratio (PER). Fish were anesthetized with MS-222 (30 mg/L), then three fish per tank were selected randomly for the measurement of body weight and body length individually to calculate condition factor (CF). The blood was taken from caudal vein and centrifuged at 3,000 rpm for 10 (4°C), then the supernatant was stored at –80°C for serum measurement. After blooding, the three fish were rapidly dissected to measure viscera and liver weight for calculating viscerosomatic index (VSI) and hepatosomatic index (HSI), respectively. The anterior intestines (1–2 cm) were sampled and immersed in Bouin’s solution for histology observation. The dorsal muscle from the left side of the body was collected and stored at –80°C for determining flesh proximate composition, collagen content, amino acids, fatty acids and biochemical parameters. Three blocks of flesh about 2–3 g were sampled from dorsal muscle on the right side of the body to determine water-holding capacity immediately (two blocks) and to store in fixative solution for tissue section observation. In addition, two blocks of dorsal muscle (1 cm^3^ and 2 cm × 2 cm × 1 cm) on the right side of the body were collected to perform the texture profiles analysis and shear force, respectively. Another two fish were taken from each tank, and the white dorsal muscle on the left was sampled in an enzyme-free tube and placed in liquid nitrogen immediately, then transferred to –80°C for metabolomics analysis (only TF group and SF group).

### Measurement indicators and methods

#### Growth performance and body morphometric indices

WG (%) = 100 × [final weight (g)—initial weight (g)]/initial weight (g)

FCR = feed intake (g)/weight gain (g)

Survival (%) = 100 × (final number of fish/initial number of fish)

FI (g/per fish) = feed intake (g)/[(final fish number + initial fish number)/2]

PER (%) = [final body weight (g)—initial body weight (g)]/[feed intake (g) × feed crude protein (%)]

VSI (%) = 100 × [final visceral weight (g)/final body weight (g)]

HSI (%) = 100 × [final liver weight (g)/final body weight (g)]

CF (g cm–^3^) = 100 × [final body weight (g)/body length (cm)^3^].

#### The diet and flesh proximate composition

The moisture, ash, crude lipid and crude protein contents in diets and flesh were analyzed following the method of AOAC (21). The moisture content was determined by drying samples to a constant weight at 105°C in a drying oven. The crude protein content (N × 6.25) was measured using the Kjeldahl system method (2,300 Auto analyZer; FOSS Tecator, AB, Hoganas, Sweden). The crude lipid content was estimated following the chloroform-methanol method. Ash content was analyzed by combusting samples in muffle furnace at 550°C for 6 h.

#### Determination of flesh amino acid, free amino acid and fatty acid

The amino acids of diets and flesh were determined using chromatography method (S-433D, Sykam, Munich, Germany) after acid hydrolysis (6 M HCl) at 110°C for 24 h. The sample was hydrolyzed using performic acid (2 mL) at 55°C for 15 min to determine methionine, then the hydrolyzed product was injected into a sodium exchange column for analysis.

The composition of free amino acid was determined according to the method described by Yu et al. (22) with a slight modification. The fresh flesh samples (0.5 g) were homogenized with 10 mL of 5% trichloroacetic acid for 1 min and stored at 4°C for 2 h. After centrifuging at 10,000 rpm for 10 min (4°C), pH was adjusted to 2 with 6 mol/L NaOH, and the supernatants were diluted to 10 mL. One milliliter of extract was filtered using a 0.22 μm membrane filter and applied to an automatic amino acid analyzer (Hitachi LA8080 Series; Japan).

The fatty acids profile of flesh was prepared by transmethylation using boron trifluoride according to the method described by Xu et al. (23) and analyzed with GC-MS (7980B gas chromatograph-mass spectrometer, Agilent Technologies).

#### Flesh quality analysis

The light (L*), redness (a*) and yellowness (b*) of flesh were measured at three locations on the cut surface of dorsal muscle using a Minolta Chroma meter (CR-10, Konica Minolta Sensing, Inc., Osaka, Japan). Water-holding capacity including the drip loss (at the 6th, 12th, 24th h) and steaming loss, was determined following the methods reported by Yang et al. (24).

The texture of the dorsal muscle, including hardness, springiness, chewiness and cohesiveness, was performed with the texture profiles analysis (TPA) using a Universal TA device (Tengba Company). Test conditions were as follows: a 25 mm × 25 mm flat-bottomed cylindrical probe, a compression ratio of 30%, a test speed of 1 mm/s and a post-test speed of 1 mm/s with the staying time of 2 s. Test conditions of shear force were as follows: Compression distance of 20 mm, a test speed of 1 mm/s and a post-test speed of 1 mm/s with a staying time of 2 s.

Total collagen, heat-soluble collagen contents of muscle were measured using hydroxyproline (Hyp) kits (Nanjing Jiancheng Bioengineering Institute, Nanjing, China). The collagen content was calculated by multiplying the Hyp content by 8 (25). Soluble collagen was extracted according to the method of Yang et al. (24). Flesh heat-soluble collagen was extracted with Ringer’s solution (32.8 mM NaCl, 1.5 mM KCl, and 0.5 mM CaCl_2_), and then measured using the method of hydroxyproline (Hyp).

Heat-insoluble collagen content = Total collagen content—heat-soluble collagen content.

#### Serum, muscle biochemical parameters analysis

Serum triglyceride (TG), cholesterol (CHO), total protein (TP) levels were assayed using Redu automatic biochemical analyzer (Chemray800, Shenzhen).

The muscle samples (0.4 g) were homogenized (1:9 w/v) in ice-cold physiological saline, and then centrifuged for 20 min at 4°C (3,000 r/min), then the supernatant was preserved at –20°C for biochemical analysis. Protein concentration was determined using the Coomassie Blue method. The total antioxidant capacity (T-AOC, Colorimetric method), superoxide dismutase (SOD, Xanthine oxidase method), glutathione peroxidase (GSH-Px, Colorimetric method), cathepsin B (CTSB, ELISA) activities and the lactate (LA) level in flesh were measured by kits (Nanjing Jiancheng Bioengineering Institute, Nanjing, China).

#### Intestine and flesh histology

The anterior intestines (about 1 cm) stored in Bouin’s solution (saturated picric acid aqueous solution: formaldehyde: glacial acetic acid = 15:5:1) and flesh (about 1 cm^3^) stored in GD fixative solution (formalin, glacial acetic acid, ethanol) were transferred to 100% ethanol, then dehydrated in graded ethanol, embedded in paraffin, sectioned (6 μm, Leica RM2235 microtome, Germany), stained with hematoxylin-eosin (HE) and sealed. Tissue sections were observed and photographed using an image microscope (Nikon YS100). Intestinal villus height, villus width, muscular thickness and muscle fiber diameter (mm^2^) and fiber density were measured using image analysis software (Image 14.0).

#### Relatively quantitative analysis of flesh metabolomics

The flesh sample stored at –80°C was taken out and placed on dry ice. Then, 15 mg flesh sample was put in an Eppendorf tube (2 mL). The flesh samples were homogenized using the homogenizer with 200 μL of H_2_O and five ceramic beads. After the addition of 800 μL methanol/acetonitrile (1:1, v/v), the homogenate was centrifuged for 15 min (14,000 g, 4°C), then the supernatant was collected and dried in a vacuum centrifuge for further use.

For LC-MS analysis, the samples were re-dissolved in 100 μL of acetonitrile/water (1:1, v/v), then vortexed and centrifuged at 14,000 g and 4°C for 15 min. Metabolic profiling of muscle was performed using an ultra-high-performance liquid chromatography (UHPLC) (1,290 Infinity LC, Agilent Technologies) equipped with a HILIC column (ACQUITY UPLC BEH Amide, 2.1 mm × 100 mm, 1.7 μm, Waters) and coupled to a quadrupole time-of-flight (AB SciexTriple TOF 6600) in Shanghai Applied Protein Technology Co., Ltd. The raw MS data (wiff.scan files) were converted to MzXML files using ProteoWizard MSConvert and processed using XCMS, including feature detection, retention time correction, and alignment. The data extracted by XCMS were performed metabolite structure identification, data preprocessing, experimental data quality evaluation and data analysis.

The data were normalized and imported into SIMCA-P for multivariate data analysis. Metabolites with the VIP value > 1 and *p*-value < 0.05 were considered as statistical significance. The metabolites were subsequently mapped to pathways in kyoto encyclopedia of genes and genomes (KEGG) for bioinformatics analysis.

### Statistical analysis

The experimental data were presented as the means ± standard deviation (means ± SD) and analyzed using SPSS 17.0 statistical software. All data were subjected to one-way analyses of variance (ANOVA) and Tukey’s multiple range tests to determine the statistical significance among treatments. Statistical significance was determined at *P* < 0.05.

## Results

### Growth performance

There was no significant difference in WG among all the groups (*P* > 0.05). Compared to TF group, FI and FCR based on dry matter were significantly decreased (*P* < 0.05), while PER was increased in SF and CF groups (*P* < 0.05). VSI and HIS in SF group and HSI in CF group were significantly higher than those in TF group (*P* < 0.05). No significant differences were detected in CF among all the groups (*P* > 0.05) (Table 3).

**TABLE 3:** Growth performance and morphologic indexes of largemouth bass fed trash fish and formula feeds.^1^

Parameters	TF	CF	SF
IBW (g)	74.9 ± 0.2	75.0 ± 0.3	75.1 ± 0.0
FBW (g)	271.2 ± 16.1	256.2 ± 2.9	266.3 ± 2.6
WG (%)	262.0 ± 20.8	242.2 ± 3.4	254.6 ± 3.6
FCR	1.09 ± 0.04^b^ (4.15 ± 0.16^b^)*	0.96 ± 0.01^a^	0.88 ± 0.02^a^
FI (g/per fish, DM)	213.9 ± 10.5^b^ (812.6 ± 39.9^b^)*	174.3 ± 0.4^a^	168.8 ± 7.1^a^
SR (%)	100	100	100
PER (%)	137.6 ± 5.3^a^	194.4 ± 2.6^b^	207.0 ± 5.9^b^
VSI (%)	7.44 ± 0.58^a^	7.74 ± 0.47^ab^	8.46 ± 0.78^b^
HIS (%)	0.98 ± 0.10a	1.78 ± 0.10^b^	2.05 ± 0.22^b^
CF (g cm^–3^)	2.47 ± 0.14	2.48 ± 0.15	2.31 ± 0.14

^1^Values are mean ± SD (*n* = 3) of three replicates, and different superscripts in the same row indicate significant differences (*P* < 0.05).

*FCR and feed intake within parentheses were based on fresh weight.

### Proximate composition and collagen content in the flesh

There were no significant differences in the contents of flesh moisture, crude protein lipid, ash and total collagen (*P* > 0.05). SF and CF groups showed significantly lower flesh heat-soluble collagen and higher heat-insoluble collagen contents than TF group (*P* < 0.05) (Table 4).

**TABLE 4:** Flesh composition of largemouth bass fed trash fish and formula feeds (fresh tissue, g/kg).^1^

Parameters	TF	CF	SF
Moisture	759.9 ± 4.9	760.5 ± 13.4	761.4 ± 1.8
Crude protein	216.7 ± 0.9	221.6 ± 12.8	217.0 ± 1.1
Crude lipid	26.0 ± 2.4	22.3 ± 1.6	22.2 ± 0.2
Ash	13.1 ± 0.2	13.2 ± 0.9	13.5 ± 0.2
Total collagen	2.50 ± 0.16	2.22 ± 0.24	2.55 ± 0.20
HS collagen	1.47 ± 0.12^b^	1.01 ± 0.06^a^	1.02 ± 0.05^a^
HIS collagen	1.03 ± 0.12^a^	1.21 ± 0.06^b^	1.52 ± 0.05^c^

^1^Values are mean ± SD (*n* = 3) of three replicates, and different superscripts in the same row indicate significant differences (*P* < 0.05).

HS collagen, Heat-soluble collagen; HIS collagen, Heat-insoluble collagen.

### Flesh color, water-holding capacity and texture

In Table 5, the yellowness (b*) in dorsal muscle of SF group was significantly higher than that of CF and TF groups (*P* < 0.05). No significant differences were observed in dorsal muscle light (L*) and redness (a*) among all the groups (*P* > 0.05). Compared to TF group, the drip loss (6, 12, and 24 h) of SF group was significantly decreased (*P* < 0.05). There was no significant difference in drip loss between TF group and CF group, and steaming loss also showed no significant difference among the three groups (*P* > 0.05).

**TABLE 5:** Flesh color, water-holding capacity and texture characteristics of largemouth bass fed trash fish and formula feeds.^1^

Parameters	TF	CF	SF
Lightness (L*)	37.61 ± 2.05	38.02 ± 1.33	38.97 ± 0.89
Redness (a*)	–2.04 ± 0.15	–2.09 ± 0.19	–2.14 ± 0.18
Yellowness (b*)	7.74 ± 0.69^a^	7.70 ± 0.51^a^	8.75 ± 0.94^b^
Drip loss ^6h^ (%)	10.09 ± 1.09^b^	10.87 ± 0.89^b^	8.31 ± 1.12^a^
Drip loss ^12^ ^h^ (%)	12.04 ± 0.75^b^	11.00 ± 0.45^ab^	10.05 ± 1.29^a^
Drip loss ^24^ ^h^ (%)	23.37 ± 2.99^b^	22.69 ± 1.49^ab^	19.09 ± 0.69^a^
Steaming loss (%)	20.55 ± 1.93	20.66 ± 2.02	20.18 ± 1.63
Hardness (gf)	310.0 ± 14.0^a^	325.3 ± 21.8^ab^	354.9 ± 29.2^b^
Springiness	0.52 ± 0.03	0.51 ± 0.04	0.54 ± 0.04
Cohesiveness (gf)	0.65 ± 0.03^b^	0.63 ± 0.04^ab^	0.57 ± 0.04^a^
Chewiness (gf)	107.8 ± 9.5	107.2 ± 11.0	111.4 ± 13.3
Shear force (gf)	1750.3 ± 158.4^a^	2073.0 ± 149.5^b^	2000.0 ± 157.1^b^

^1^Values are mean ± SD (*n* = 3) of three replicates, and different superscripts in the same row indicate significant differences (*P* < 0.05).

Both CF and SF groups showed significantly higher shear force than TF group (*P* < 0.05), and SF group also presented higher flesh hardness and lower cohesiveness than TF group (*P* < 0.05). There were no significant differences in the flesh chewiness and springiness among all the groups (*P* > 0.05).

### Flesh amino acid and fatty acid

There were no significant effects of feeding trash fish, commercial feed and self-made feed on flesh total amino acids, essential amino acids of largemouth bass (*P* > 0.05) (Table 6). Flesh isoleucine content of SF group was significantly lower than that of CF and TF groups (*P* < 0.05).

**TABLE 6:** Flesh amino acid composition of largemouth bass fed trash fish and formula feeds (DM basis, g/kg).^1^

	TF	CF	SF
Aspartic acid	94.0 ± 1.9	96.1 ± 2.0	97.5 ± 1.1
Threonine	42.1 ± 1.0	41.5 ± 0.4	42.0 ± 0.9
Serine	37.9 ± 0.7	37.5 ± 0.8	38.9 ± 1.4
Glutamic acid	132.3 ± 2.9	133.9 ± 2.0	135.2 ± 1.8
Glycine	37.9 ± 0.1	39.1 ± 0.8	41.5 ± 2.2
Alanine	55.2 ± 1.1	52.4 ± 0.9	54.5 ± 1.7
Cysteine	6.6 ± 0.3	6.7 ± 0.1	6.0 ± 0.8
Valine	38.7 ± 1.7	36.1 ± 1.6	35.3 ± 0.3
Methionine	23.9 ± 2.1	22.9 ± 2.1	24.6 ± 1.4
Isoleucine	35.8 ± 0.0^b^	32.8 ± 2.2^ab^	28.5 ± 1.1^a^
Leucine	78.2 ± 1.6	78.7 ± 0.8	78.2 ± 2.8
Tyrosine	31.5 ± 0.6	33.3 ± 1.6	33.4 ± 1.2
Phenylalanine	41.0 ± 0.2	44.8 ± 3.7	43.6 ± 1.5
Lysine	84.5 ± 1.8	87.5 ± 1.9	84.1 ± 4.4
Histidine	23.0 ± 1.4	25.2 ± 1.7	21.6 ± 0.6
Arginine	53.6 ± 1.5	53.8 ± 0.4	53.1 ± 2.5
Proline	26.3 ± 0.6	26.4 ± 0.5	26.4 ± 0.2
EAAs	420.7 ± 7.5	423.4 ± 6.8	411.1 ± 11.6
TAAs	842.4 ± 13.5	848.8 ± 9.2	844.5 ± 10.8

^1^Values are mean ± SD (*n* = 3) of three replicates, and different superscripts in the same row indicate significant differences (*P* < 0.05).

EAAs, essential amino acids; TAAs, total amino acids.

Seventeen free amino acids were detected in the flesh. Histidine had the highest content, followed by glycine. Compared to TF group, the contents of total free amino acids (TFAAs) and essential amino acids (EAAs) in SF and CF groups were significantly decreased, and the free flavor amino acids (FAAs) content of SF group were significantly increased (*P* < 0.05). SF and CF groups had lower contents of flesh aspartic acid, threonine, serine, glutamic acid, methionine, isoleucine, leucine, lysine, histidine and arginine, and higher glycine and valine contents than TF group (*P* < 0.05). Compared to CF group, SF group showed higher TFAAs, DAAs, threonine, serine, glutamic acid, alanine and proline contents and lower lysine content (*P* < 0.05) (Table 7).

**TABLE 7:** Flesh free amino acid composition of largemouth bass fed trash fish and formula feeds (wet weight, mg/kg).^1^

	TF	CF	SF
Aspartic acid*	24.1 ± 1.2^b^	20.8 ± 0.7^a^	20.8 ± 1.4^a^
Threonine	387.1 ± 29.3^c^	110.9 ± 9.7^a^	207.5 ± 11.8^b^
Serine	174.4 ± 8.6^c^	58.3 ± 3.9^a^	86.7 ± 7.6^b^
Glutamic acid*	153.4 ± 13.3^c^	77.3 ± 5.5^a^	128.1 ± 6.0^b^
Glycine*	517.5 ± 51.8^a^	711.5 ± 45.1^b^	805.1 ± 42.1^b^
Alanine*	301.2 ± 28.8^b^	196.5 ± 17.7^a^	265.2 ± 13.6^b^
Cysteine	18.5 ± 1.9	17.2 ± 1.7	17.0 ± 1.7
Valine	91.8 ± 1.8^a^	101.8 ± 4.3^b^	101.7 ± 0.9^b^
Methionine	83.8 ± 2.8^b^	62.4 ± 0.6^a^	64.4 ± 2.4^a^
Isoleucine	34.8 ± 2.8^b^	22.0 ± 1.5^a^	23.0 ± 1.0^a^
Leucine	62.2 ± 4.5^b^	39.4 ± 2.5^a^	42.7 ± 0.2^a^
Tyrosine	91.1 ± 8.8^b^	71.0 ± 4.5^a^	75.9 ± 6.9^ab^
Phenylalanine	141.8 ± 15.3	125.4 ± 11.3	154.8 ± 12.9
Lysine	302.5 ± 24.9^c^	224.9 ± 22.2^b^	147.6 ± 11.1^a^
Histidine	1433.2 ± 108.0^b^	1071.3 ± 10.9^a^	890.8 ± 66.2^a^
Arginine	42.4 ± 4.3^b^	26.1 ± 2.2^a^	25.0 ± 2.2^a^
Proline	299.4 ± 22.5^b^	83.2 ± 8.3^a^	261.8 ± 15.1^b^
FAAs	996.2 ± 86.5^a^	1006.1 ± 60.7^a^	1219.2 ± 53.1^b^
EAAs	2579.7 ± 106.0^b^	1784.0 ± 51.3^a^	1657.6 ± 87.8^a^
TFAAs	4159.2 ± 140.1^c^	3019.7 ± 100.1^a^	3318.2 ± 82.1^b^

^1^Values are mean ± SD (*n* = 3) of three replicates, and different superscripts in the same row indicate significant differences (*P* < 0.05).

FAAs, flavor amino acids (*); EAAs, essential amino acids; TFAAs, total free amino acids.

The most abundant fatty acid was polyunsaturated fatty acids (PUFAs) (42–55%), followed by monounsaturated fatty acids (MUFAs) (27–39%) and saturated fatty acids (SFAs) (18–20%). Compared to TF group, SF and CF groups had significantly lower MUFAs level and higher PUFAs, n-3 PUFAs and n-6 PUFAs levels (*P* < 0.05). Higher C18:2, C20:5, C22:6 level and lower C14:0, C17:0, C16:1, C18:1, C18:3, C20:1, C20:3, C20:4, and C22:5 levels were observed in SF and CF groups than those in TF group (*P* < 0.05). C20:1 and C20:5 levels in SF group were significantly higher than those in CF group (*P* < 0.05) (Table 8).

**TABLE 8:** Flesh fatty acid composition of largemouth bass fed trash fish and formula feeds (percentage of fatty acids,%).^1^

	TF	CF	SF
C14: 0	1.38 ± 0.07^b^	1.02 ± 0.03^a^	1.10 ± 0.07^a^
C16: 0	14.17 ± 0.25	13.37 ± 0.16	13.58 ± 0.44
C17: 0	0.45 ± 0.01^b^	0.20 ± 0.01^a^	0.22 ± 0.01^a^
C18: 0	3.74 ± 0.29	4.10 ± 0.06	3.74 ± 0.11
C16: 1	6.90 ± 0.51^b^	3.05 ± 0.14^a^	3.09 ± 0.37^a^
C18: 1	29.84 ± 0.13^b^	24.53 ± 1.49^a^	22.68 ± 1.76^a^
C20: 1	1.43 ± 0.06^c^	0.93 ± 0.01^a^	1.29 ± 0.01^b^
C18: 2	14.22 ± 0.51^a^	22.31 ± 0.62^b^	20.81 ± 0.87^b^
C18: 3	3.29 ± 0.24^b^	2.08 ± 0.05^a^	1.75 ± 0.05^a^
C20: 2	1.10 ± 0.02	1.13 ± 0.06	1.08 ± 0.03
C20: 3	0.77 ± 0.03^b^	0.26 ± 0.02^a^	0.26 ± 0.01^a^
C20: 4	4.06 ± 0.06^b^	1.78 ± 0.09^a^	1.75 ± 0.15^a^
C20: 5 (EPA)	1.97 ± 0.00^a^	2.61 ± 0.11^b^	3.85 ± 0.30^c^
C22: 5	4.94 ± 0.03^b^	2.89 ± 0.11^a^	3.15 ± 0.16^a^
C22: 6 (DHA)	11.73 ± 0.70^a^	19.74 ± 1.19^b^	21.67 ± 0.99^b^
SFAs	19.74 ± 0.48	18.69 ± 0.22	18.64 ± 0.40
MUFAs	38.16 ± 0.44^b^	28.51 ± 1.60^a^	27.06 ± 2.14^a^
PUFAs	42.09 ± 0.03^a^	52.79 ± 1.69^b^	54.31 ± 2.54^b^
n-3 PUFAs	21.93 ± 0.43^a^	27.32 ± 1.40^b^	30.41 ± 1.49^b^
n-6 PUFAs	20.16 ± 0.46^a^	25.48 ± 0.57^b^	23.89 ± 1.05^b^

^1^Values are mean ± SD (*n* = 3) of three replicates, and different superscripts in the same row indicate significant differences (*P* < 0.05).

SFAs, saturated fatty acids; MUFAs, monounsaturated saturated fatty acids; PUFAs, polyunsaturated fatty acids.

### Serum and muscle biochemical parameter

In serum, TP, TG and CHO contents in SF group were significantly lower than those in TF and CF groups (*P* < 0.05). In flesh, there were no significant effects of feeding trash fish, commercial feed and self-made feed on LA content, T-AOC, SOD, GSH-Px and CTSB activities (*P* > 0.05) (Table 9).

**TABLE 9:** Serum and flesh biochemical indices of largemouth bass fed trash fish and formula feeds.^1^

Parameters	TF	CF	SF
**Serum**			
TP (g/L)	42.73 ± 1.16^b^	47.98 ± 3.47^b^	30.71 ± 2.28^a^
TG (mmol/L)	5.11 ± 0.01^b^	4.77 ± 0.57^b^	2.75 ± 0.21^a^
CHO (mmol/L)	12.91 ± 1.13^b^	12.43 ± 0.41^b^	6.06 ± 0.09^a^
**Muscle**			
T-AOC (U/mg prot)	1.53 ± 0.05	1.57 ± 0.05	1.48 ± 0.07
SOD (U/mg)	19.75 ± 0.92	20.24 ± 1.02	19.25 ± 0.16
GSH-Px (U/mg)	38.46 ± 1.04	39.40 ± 1.57	38.06 ± 1.00
LA (nmol/mg)	6.29 ± 0.21	6.21 ± 0.32	6.44 ± 0.29
CTSB (ng/g)	100.6 ± 4.4	108.5 ± 5.0	105.2 ± 1.8

^1^Values are mean ± SD (*n* = 3) of three replicates, and different superscripts in the same row indicate significant differences (*P* < 0.05).

TP, total protein; TG, triglyceride; CHO, cholesterol; T-AOC, total antioxidant capacity; SOD, superoxide dismutase; GSH-Px, glutathione peroxidase; CTSB, cathepsin B.

### Muscle and intestine histology

**FIGURE 1:**
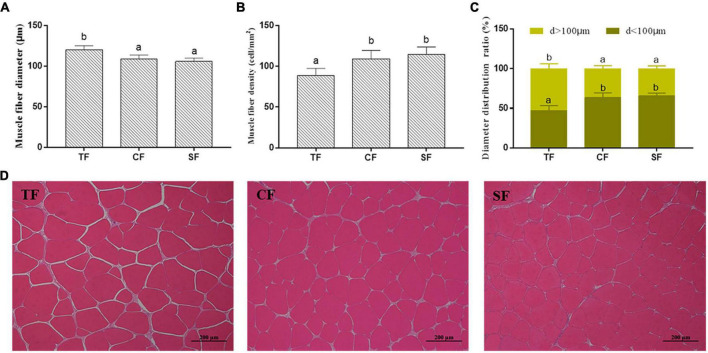
The flesh structure of largemouth bass fed trash fish and formula feeds. Values are means ± SD (*n* = 3) of three replicates. Bars with different letters denote significant difference among treatments (*P* < 0.05). **(A–C)** Muscle fiber density (cell/mm^2^), Muscle fiber diameter (μm) and Muscle fiber diameter distribution ratio (%), **(D)** Histological structure (100×).

Higher muscle fiber density and lower muscle fiber diameter were observed in SF and CF groups than those in TF group (*P* < 0.05). The muscle cells of SF group arranged closely with smaller intercellular spaces, while TF group showed larger intercellular spaces (Figure 1).

**FIGURE 2:**
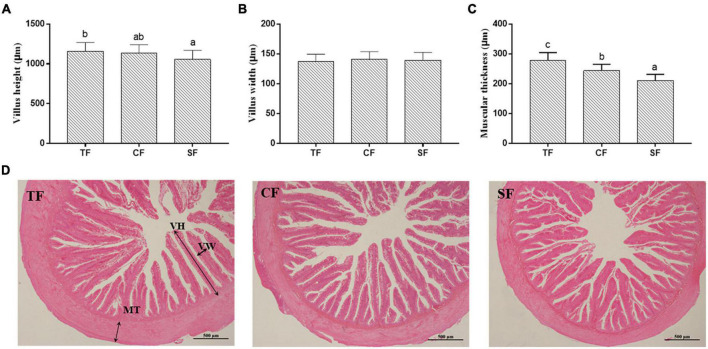
The intestinal structure of largemouth bass fed trash fish and formula. Values are means ± SD (*n* = 3) of three replicates. Bars with different letters denote significant difference among treatments (*P* < 0.05). (**A–C**) Muscle fiber density (cell/mm^2^), Muscle fiber diameter (μm), Muscle fiber diameter distribution ratio (%), **(D)** Histological structure (100×). VH, villus height; VW, villus width; MT, muscular thickness.

In intestinal histology, all the groups showed integral morphology, and the intestinal villus height in SF group and muscular thickness in SF and CF groups were significantly lower than those in TF group (*P* < 0.05) (Figure 2).

### Relatively quantitative analysis of flesh metabolomics

#### Identification of differential metabolites

**FIGURE 3:**
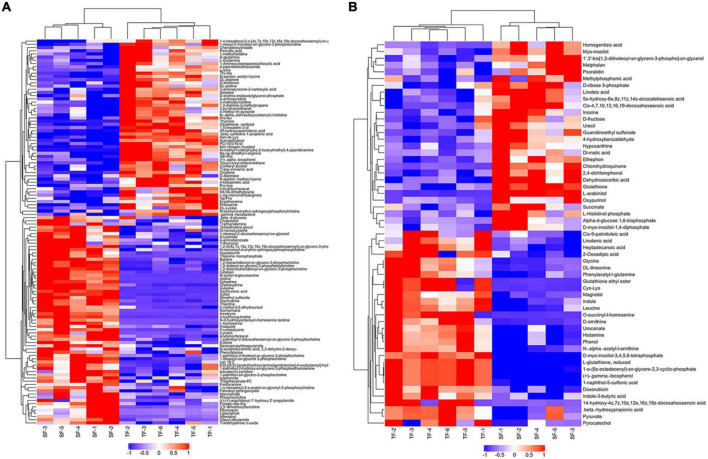
Hierarchical clustering diagram of metabolites with significant differences between TF and SF group. TF-1 to TF-6: Trash fish group; SF-1 to SF-5: Self-made feed group. Red represented the up-regulated substance, blue represented the down-regulated substance. **(A)** Positive ion mode; **(B)** Negative ion mode.

There were 1,041 metabolites identified, including 568 metabolites in the positive (POS) ion mode and 473 metabolites in the negative (NEG) ion mode between SF and TF groups. Metabolites with multivariate (VIP value > 1) and univariate (*p*-value < 0.05) statistical significance criteria were considered as statistical significance. In the metabolites comparison of SF vs. TF, a total of 177 significantly differential metabolites were screened, including 120 (65 up-regulated, 55 down-regulated) in the positive ion mode and 57 (28 up-regulated, 29 down-regulated) in the negative ion mode (Supplementary Table 1). The main differential metabolites were organic acids and derivatives (59 types), lipids and lipid-like molecules (35 types) according to Class classification. They were mainly focused on carboxylic acids and derivatives (threonine, lysine, leucine, histidine, glycine, glutathione, etc.), fatty acyls (20-hydroxyarachidonic acid, heptadecanoic acid, cis-4, 7, 10, 13, 16, 19-docosahexaenoic acid, linoleic acid, linolenic acid, cis-9-palmitoleic acid, etc.), glycerophospholipids (1,2-diarachidonoyl-sn-glycero-3-phosphocholine, 1,2- didocosahexaenoyl-sn-glycero-3-phosphocholine, 1-palmitoyl- 2-docosahexaenoyl-sn-glycero-3-phosphocholine, 1-palmitoyl- 2-linoleoyl-sn-glycero-3-phosphocholine, etc.) (Figure 3).

#### Kyoto encyclopedia of genes and genomes pathway analysis of differential metabolites

**FIGURE 4:**
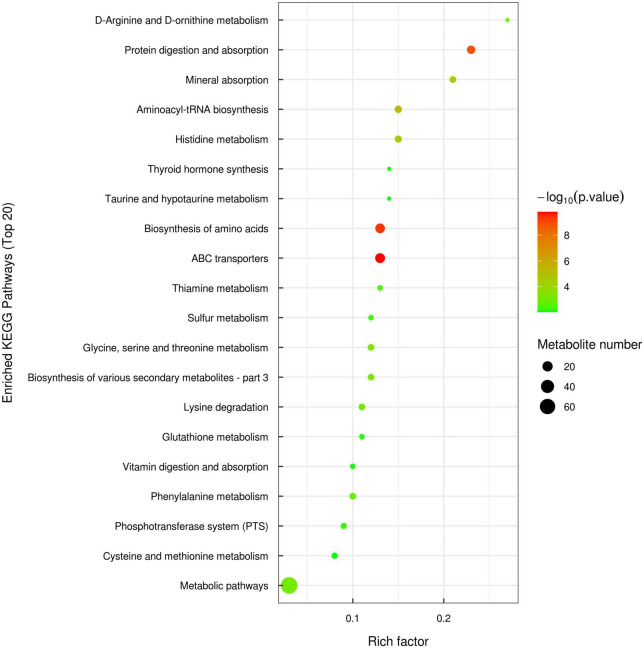
Bubble diagram of kyoto encyclopedia of genes and genomes (KEGG) metabolic pathway enrichment analysis of significantly different metabolites between TF and SF group (top 20).

The identified differential metabolites were assigned to the KEGG database,^1^ and a total of 119 differential metabolites were annotated to amino acid metabolism, carbohydrate metabolism, digestive system, nucleotide metabolism and energy metabolism, etc. The KEGG enrichment analysis suggested that 119 differential metabolites were enriched in 37 types of metabolic pathways. Among them, signaling pathways related to amino acid metabolism were the most abundant and consisted of 15 types, including biosynthesis of amino acids, histidine metabolism, glycine, serine and threonine metabolism, arginine biosynthesis, phenylalanine metabolism, etc. In addition, ABC transporters, protein digestion and absorption, mineral absorption, lysine degradation and glutathione metabolism were also significantly enriched. The top 20 metabolic pathways with the most enriched differential metabolites were selected to make a bubble diagram (Figure 4).

## Discussion

### Growth performance and feed utilization

Largemouth bass is typically carnivorous fish with high requirement for dietary protein. In the present study, the crude protein contents of trash fish, self-made feed and commercial feed were 66.62, 54.73, and 53.53% at dry matter basis, respectively. However, there was no significant difference in weight gain of largemouth bass among the three groups, and the weight gain of TF group was just numerically higher. In addition, the protein efficiency ratio of TF group was significantly decreased, indicating that the protein level of the formula feeds can sufficiently meet the growth needs of largemouth bass, and the surplus protein would be used for other purposes. Bunlipatanon et al. (26) found that Asian sea bass (*Latescal carifer*) and tiger grouper (*Epinephelus fuscoguttatus*) fed with pellet feeds had similar growth performance and survival to the fish fed with trash fish. Similar result was also reported in European sea bass (*Dicentrarchus labrax*) (27). However, Nugraha and Rozi (28) reported that grouper fed commercial feed had higher specific growth rate, survival, feed utilization efficiency and lower feed conversion ratio than those fed trash fish. Maybe trash fish is easy to pollute the water quality due to the dissolution and diffusion of some components in flesh blocks, resulting in a high risk of sanitation and pathogen transmission, thereby reducing growth performance. In this study, recirculating aquaculture was adopted to keep the same water quality in each group, and no feed residue left after feeding. So, the growth results depended on the quality of feed. Remarkably, formula feeds contain some component that cannot be fully digested such as non-starch polysaccharides, and its digestibility is lower than that of trash fish. However, the weight gain of formula feeds groups was not significantly different from that of TF group, and the FCR and protein efficiency ratio were even better than TF group. The present results suggested that the commercial feed and self-made feed could completely replace trash fish during the 12-week feeding period without adverse effects on the growth performance. Furthermore, formula feeds improved the feed utilization with lower FCR, which would be helpful to maintain good water quality in aquaculture.

Morphological index can reflect the energy reserve and nutritional status of fish. In this study, the HSI and VSI in SF group were significantly higher than those in TF group. Such result was consistent with the studies in humpback grouper (*Cromileptes altivelis*) (29), hybrid grouper (*Epinephelus fuscoguttatus* × *Epinephelus lanceolatus*) (30) and largemouth bass (4). The higher carbohydrate content in formula feed than in trash fish may change glucose metabolism and lead to the glycogen deposition in liver, resulting in the increase of HSI and VSI.

### Nutritional value and flavor of flesh

The type, quantity and composition of amino acids are the indicators in fish nutrition evaluation. In this study, there was no significant difference in the flesh crude protein and amino acid contents between TF group and formula feed groups. Free amino acid composition of flesh plays an important role in flavor. Alanine, glutamic acid, aspartic acid and glycine are the main flavor amino acids (31), in which glutamic acid and aspartic acid contribute to the umami taste, while alanine and glycine are crucial to the sweetness taste (32). High flavor amino acids and essential amino acids contents can improve flesh flavor and quality in fish (33). In this study, TFAAs and EAAs contents in SF and CF groups were significantly lower than those in TF group, while the contents of FAAs and glycine in SF group were significantly higher than those in TF group, indicating that flesh sweetness in SF group may be better than that in TF group. TF group showed higher levels of aspartic acid and glutamic acid, which contributed to the umami taste of flesh. In metabolomics analysis, amino acid metabolites in flesh between SF group and TF group changed significantly. Leucine, threonine, histidine, arginine, lysine and proline were significantly down-regulated, which was consistent with the changes of free amino acid contents in flesh. Trash fish contains plenty of active substances as nucleotides, taurine, free amino acids, peptides and unknown growth factors, and these components may increase the free amino acid content in flesh by regulating amino acid transport. Remarkably, the formula feed groups showed lower histidine content than TF group. In flesh metabolomics, the down-regulation of histidine in SF group was involved in histidine metabolism, biosynthesis of amino acid, protein digestion and absorption, ABC transporter, etc. With the action of microbial histamine decarboxylase, free histidine in fish flesh was converted to histamine, and excessive histamine deposition would cause meat deterioration (34). From this point, largemouth bass fed with formula feed may have better shelf life of flesh than the fish fed with trash fish.

Flesh fatty acid content and composition of fish are easily affected by diet (35). In the present study, significant differences were observed in the flesh fatty acid content between formula feeds groups and TF group, which may be caused by the difference of fatty acid composition in feeds. The PUFAs levels including C20:5 (EPA) and C22:6 (DHA) were higher in formula feeds than those in trash fish, leading to higher flesh EPA and DHA ratios of largemouth bass fed formula feeds. Such a result was consistent with the reports in large yellow croaker by Ma et al. (11) and in largemouth bass by Plaipetch et al. (14). In the comparison of flesh metabolomics of SF group vs. TF group, cis-9-palmitoleic acid (C16:1), heptadecanoic acid (C17:0) and linolenic acid (C18:3) were down-regulated, and linoleic acid (C18:2) and cis-4, 7, 10, 13, 16, 19-docosahexaenoic acid (C22:6, DHA) were up-regulated, which were mainly involved in the biosynthesis of unsaturated fatty acids, linoleic acid metabolism and alpha-linolenic acid metabolic pathways. Normally, the ingested linolenic acid can be converted to eicosapentaenoic acid (EPA) under the role of various enzymes such as dehydrogenase and carbon chain elongase, and then produce docosahexaenoic acid (DHA) *via* β-oxidation (36). EPA and DHA play important roles to human health in reducing blood lipid, enhancing the toughness of blood vessel, anti-aging and promoting brain development (37). It indicated that the fatty acid composition of artificial formula feed is more balanced and more suitable for improving the flesh nutritional value of fish than trash fish.

### Flesh physical and chemical characteristics

In this study, largemouth bass of SF group showed higher flesh yellowness than CF and TF groups, which may be related to the inclusion of corn gluten meal in SF diet. Corn gluten meal is rich in carotenoids and easy to deposit in flesh. The flesh hardness and shear force in formula feed groups were significantly higher than those in TF group, which was consistent with the results in large yellow croaker (11). The reason might be related to muscle fibers and collagen content. Previous studies have shown that flesh hardness is negatively correlated with muscle fiber diameter and positively correlated with muscle fiber density (38, 39). Compared to TF group, muscle fiber diameters in formula feed groups were decreased, and muscle fiber densities were increased with smaller muscle fiber interstice, which enhanced the intercellular binding ability. Collagen is the major protein component in intramuscular connective tissues with the function of stabilizing muscle structure and integrity. Flesh hardness depends on the content, heat solubility, crosslink degree and thermal stability of collagen (40). In this study, there was no significant difference in the flesh total collagen content among all the groups, while the heat-insoluble collagen contents in formula feed groups were significantly higher than that in TF group, indicating that the collagen molecules were more stable, thereby enhancing the flesh hardness. Generally, the high flesh hardness of largemouth bass fed formula feeds is more acceptable to consumers in the market.

Water-holding capacity directly influences the flesh taste, juiciness, tenderness, color, flavor and the yield and quality of meat (41). In the present study, flesh drip loss of SF group was significantly lower than that of TF group. May be the higher muscle fiber density and smaller muscle fiber interstice in SF group enhanced the muscle intercellular interaction, and bound more water in muscle (42, 43). Strong water holding capacity could maintain good flesh flavor and nutrients. In addition, higher lipid content of dietary trash fish is easily oxidized and damages the muscle cell membrane, thereby reducing the water-holding capacity of the muscle cells in TF group.

### Serum and flesh biochemical parameters

Serum triglyceride (TG) and cholesterol (CHO) contents were important indicators reflecting lipid metabolism in the body (44). In this study, lower serum TG and CHO contents in SF group than TF group may be due to lower lipid levels in formula feeds than trash fish. Similar result was also reported by Cao (45) in the study of snakehead. The increased TG and CHO levels in serum suggested an active endogenous lipid transportation, which is the normal response to high-lipid diets without adverse effects on liver function (46). Higher TG and CHO levels in CF group than SF group may be related to the composition of different ingredients and lipid types.

The antioxidant capacity of flesh after slaughter is a key factor affecting flesh quality (47, 48). Our results showed that no significant differences were observed in lactic acid (LA) content, total antioxidant capacity (T-AOC), superoxide dismutase (SOD), glutathione peroxidase (GSH-Px) and cathepsin B (CTSB) activities of largemouth bass among all groups, indicating that flesh antioxidant capacity was less affected by feed in the present study.

### Intestinal structure

The height and number of intestinal villus and intestinal morphology can reflect the digestive and absorptive capacity of fish intestine. Larger height and width of intestinal villus mean more contact area in favor of nutrient absorption (49). Compared to the fish fed with trash fish, mandarin fish (*Siniperca chuatsi*♀ × *Siniperca scherzeri*♂) presented lower intestinal muscle thickness (50), and largemouth bass (4), snakehead (45) showed lower intestinal villus height. In this study, the intestinal villus height and muscle thickness in formula feed groups were also significantly lower than those in trash fish group. Trash fish is easily digested and absorbed, while the formula feeds contain some components that cannot be fully digestible and absorbable for carnivorous fish, such as non-starch polysaccharides in vegetable protein sources. Maybe these components increase the intestinal filling and decrease the muscle thickness of intestinal wall.

## Conclusion

In this study, feeding trash fish, commercial feed and self-made feed did not affect the growth performance of largemouth bass. Largemouth bass fed trash fish has higher flesh free amino acid content, intestinal villus height and muscle thickness, while the fish fed formula feeds has higher levels of flesh heat-insoluble collagen and polyunsaturated fatty acid, flesh hardness and muscle fiber density, and lower drip loss and muscle fiber diameter.

## Data availability statement

The original contributions presented in this study are included in the article/Supplementary material, further inquiries can be directed to the corresponding author/s.

## Ethics statement

The animal study was reviewed and approved by the Institutional Animal Care and Use Committee of Shanghai Ocean University.

## Author contributions

XX: methodology, formal analysis, investigation, data curation, and writing—original draft preparation. HY: investigation, methodology, data curation, and writing—original draft preparation. ZX: investigation, methodology, validation, and formal analysis. XL and XJL: funding acquisition, resources, writing—review and editing, and supervision. All authors contributed to the article and approved the submitted version.

## Footnotes

1
http://www.genome.jp/kegg/pathway.html

## References

[1] Fishery administration, Ministry of Agriculture and Rural Affairs. *China Fishery Statistics Yearbook.* Beijing: Agriculture Press (2021). p. 24–5.

[2] Kim JH Gomez DK Choresca CH Park SC. Detection of major bacterial and viral pathogens in trash fish used to feed cultured flounder in Korea. *Aquaculture.* (2007) 272:105–10. 10.1016/j.aquaculture.2007.09.008

[3] Xu Z Lin X Lin Q Yang Y Wang Y. Nitrogen, phosphorus, and energy waste outputs of four marine cage-cultured fish fed with trash fish. *Aquaculture.* (2007) 263:130–41. 10.1016/j.aquaculture.2006.10.020

[4] Mou MM Jiang Y Luo Q Chen YJ Luo L Lin SM. Effects of formulated diet and fresh frozen *Hypophthalmichthys molitrix* on growth, plasma biochemical index and antioxidant ability and histology of *Micropterus salmoides*. *J Fish China.* (2018) 42:1408–16.

[5] Li ZF Gong WB Wang JL Wang GJ Yu DG Xie J. Evaluation of effects of frozen fresh fish and artifical compound feed on muscle quality and health status of *Micropterus salmoides*. *Chin J Anim Nutr.* (2017) 29:4180–8.

[6] Guan SJ Wu RQ Xie J Wang GJ Niu JF. Effects of two diets on growth, digestive index and digestive enzyme activities of largemouth bass (*Micropterus salmoides*). *Feed Ind.* (2007) 28:32–6.

[7] Yu EM Zhang ZN Xia Y Xie J Wang GJ Yu DG. Effects of different diets on intestinal microflora of largemouth bass(*Micropterus salmoides*). *J Fish China.* (2015) 39:118–26.

[8] Hien TTT Trung NHD Tâm BM Chau VMQ Huy NH Lee CM Replacement of freshwater small-size fish by formulated feed in snakehead (*Channa striata*) aquaculture: experimental and commercial-scale pond trials, with economic analysis. *Aquac Rep.* (2016) 4:42–7. 10.1016/j.aqrep.2016.06.003

[9] Zhang YH Ma GP Song LP. Effect of factory farming on feeding iced fish and formulated feed on muscle quality of *Channa argus*. *J Yangtze Univ.* (2019) 16:72–7.

[10] Li XQ Li BA Chen NS Huang XX Hua XM Leng XJ. A comparison study on flesh quality of large yellow croaker (*Larimichthys croceus*) cultured with three different modes. *J Ocean Univ China.* (2017) 16:1187–94. 10.1007/s11802-017-3338-0

[11] Ma R Men YQ Zhang WB Mai KS. Comparative study on the organoleptic quality of wild and farmed large yellow croaker *Larimichthys crocea*. *J Oceanol Limnol.* (2019) 38:260–74. 10.1007/s00343-019-8353-0

[12] Zhou PP Jin M Wu WJ ShenTu JK Li M Zhou QC. Comparison of nutrient components of large yellow croaker (*Pseudosciaena crocea* Richardson) cultured in different modes, fed different feeds and from different strains. *Chin J Anim Nutr.* (2014) 26:969–80.

[13] Niu XJ Feng HM Zhao XQ Qin YM Ye JD. The effect comparisons between frozen trash fish and artifical compound feed for grouper (*Epinephelus coioides*). *J Jimei Univ.* (2021) 26:8–13.

[14] Plaipetch P Tamtin M Chaikul SL Kuekaew J Muengyao P Samranrat N Comparison on growth performance and meat quality of sea bass fed with trash fish and pellets. *Proceedings of the 45th Kasetsart University Annual Conference.* Kasetsart: Fisheries (2008). p. 156–66.

[15] Mannina L Sobolev AP Capitani D Iaffaldano N Rosato MP Ragni P NMR metabolic profiling of organic and aqueous sea bass extracts: implications in the discrimination of wild and cultured sea bass. *Talanta.* (2008) 77:433–44. 10.1016/j.talanta.2008.07.006 18804657

[16] Maruhenda Egea FC Toledo-Guedes K Sanchez-Jerez P Ibanco-Cañete R Uglem I Saether BS. A metabolomic approach to detect effects of salmon farming on wild saithe (*Pollachius virens*) populations. *J Agric Food Chem.* (2015) 63:10717–26. 10.1021/acs.jafc.5b04765 26600204

[17] Yan J Tian LX Xie SW Guo DQ Yang HJ Liang GY Interactions between dietary protein levels, growth performance, feed utilization, gene expression and metabolic products in juvenile grass carp (*Ctenopharyngodon idella*). *Aquaculture.* (2015) 437:75–83. 10.1016/j.aquaculture.2014.11.031

[18] Wei ZH Zhou HH Zhang YJ Zhang Q Zhang WB Mai KS. Integrative analysis of transcriptomics and metabolomics profiling on flesh quality of large yellow croaker *Larimichthys crocea* fed a diet with hydroxyproline supplementation. *Br J Nutr.* (2018) 119:359–67. 10.1017/s0007114517003968 29498352

[19] Deborde C Hounoum BM Moing A Maucourt M Jacob D Corraze G Putative imbalanced amino acid metabolism in rainbow trout long term fed a plant-based diet as revealed by 1H-NMR metabolomics. *J Nutr Sci.* (2021) 10:1–18. 10.1017/jns.2021.3 33889396 PMC8057518

[20] Wei YL Liang M Mai K Zheng K Xu H. The effect of ultrafiltered fish protein hydrolysate levels on the liver and muscle metabolic profile of juvenile turbot (*Scophthalmus maximus* L.) by 1H NMR-based metabolomics studies. *Aquac Res.* (2016) 48:3515–27. 10.1111/are.13178

[21] AOAC. *Official Methods of Analysis.* 18th ed. Gaithersburg, MD: Association of Official Analytical Chemists (2005).

[22] Yu DW Xu YC Regenstein JM Xia WS Yang F Jiang QX The effects of edible chitosan-based coatings on flavor quality of raw grass carp (*Ctenopharyngodon idellus*) fillets during refrigerated storage. *Food Chem.* (2018) 242:412–20. 10.1016/j.foodchem.2017.09.037 29037708

[23] Xu XY Yang H Zhang CY Bian YH Yao WX Xu Z Effects of replacing fishmeal with cottonseed protein concentrate on growth performance, flesh quality and gossypol deposition of largemouth bass (*Micropterus salmoides*). *Aquaculture.* (2022) 548:737551. 10.1016/j.aquaculture.2021.737551

[24] Yang H Xu Z Li XQ Tan SM Cheng Z Leng XJ. Influences of dietary *Eucommia ulmoides* extract on growth, flesh quality, antioxidant capacity and collagen-related genes expression in grass carp (*Ctenopharyngodon idellus*). *Anim Feed Sci Technol.* (2021) 277:114965. 10.1016/j.anifeedsci.2021.114965

[25] AOAC. *Meat and Meat Products: Hydroxyproline in Meat and Meat Products, Method 990.26. Ch 39. Official Methods of Analysis.* 16th ed. Arlington, TX: Association of Official Analytical Chemists (1998).

[26] Bunlipatanon P Songseechan N Kongkeo H Abery NW De Silva SS. Comparative efficacy of trash fish versus compounded commercial feeds in cage aquaculture of Asian seabass (*Lates calcarifer*) (Bloch) and tiger grouper (*Epinephelus fuscoguttatus*) (Forsskål). *Aquacult Res.* (2014) 45:373–88. 10.1111/j.1365-2109.2012.03234.x

[27] El-Hammady AKI Ibrahim SA Wafa MAI El-Ghamadi FA. Feeding European sea bass (*Dicentrarchus labrax*) with trash fish 1-growth performance and reproductive histology. *Life Sci J.* (2014) 11:568–83.

[28] Nugraha MA Rozi. The effect of giving commercial feed, beloso trash fish (*Saurida tumbil*), kurisi trash fish (*Nemipterus nematophorus*), and mixed trash fish on growth of cantang grouper (*Epinephelus fuscoguttatus-lanceolatus*) in floating net cage. *IOP Conf Ser Earth Environ Sci.* (2020) 441:012069. 10.1088/1755-1315/441/1/012069

[29] Shapawi R Mustafa S Ng WK. A comparison of the growth performance and body composition of the Humpback Grouper, *Cromileptes altivelis* fed on farm-made feeds, commercial feeds or trash fish. *J Fish Aquat Sci.* (2011) 6:523–34. 10.3923/jfas.2011.523.534

[30] Cong LM Wang WF Gao CR Huang B Lei JL Wang GQ. Effects of compound diet and fresh frozen *Ammodytes personatus* on growth, antioxidant ability and lipid metabolism of hybrid grouper (*Epinephelus fuscoguttatus*♀×*Epinephelus lanceolatus*♂) juvenilles. *J Fish China.* (2016) 40: 1398–407.

[31] Ruiz-Capillas C Moral A. Free amino acids in muscle of Norway lobster (*Nephrops novergicus* (L.)) in controlled and modified atmospheres during chilled storage. *Food Chem.* (2004) 86:85–91. 10.1016/j.foodchem.2003.08.019

[32] Kawai M Okiyama A Ueda Y. Taste enhancements between various amino acids and IMP. *Chem Senses.* (2002) 27:739–45. 10.1093/chemse/27.8.739 12379598

[33] Buchtová H Svobodová Z Kocour M Velíšek J. Amino acid composition in fillets of mirror crossbreds common carp (*Cyprinus carpio*, Linnaeus 1758). *Acta Vet Brno.* (2009) 78:337–44. 10.2754/avb200978020337 22026406

[34] Yesudhason P Al-Zidjali M Al-Zidjali A Al-Busaidi M Al-Waili A Al-Mazrooei N Histamine levels in commercially important fresh and processed fish of Oman with reference to international standards. *Food Chem.* (2013) 140:777–83. 10.1016/j.foodchem.2012.11.030 23692766

[35] Xu H Turchini GM Francis DS Liang M Mock TS Rombenso A Are fish what they eat? A fatty acid’s perspective. *Prog Lipid Res.* (2020) 80:101064. 10.1016/j.plipres.2020.101064 33010278

[36] Kim KB Nam YA Kim HS Hayes AW Lee BM. alpha-Linolenic acid: nutraceutical, pharmacological and toxicological evaluation. *Food Chem Toxicol.* (2014) 70:163–78. 10.1016/j.fct.2014.05.009 24859185

[37] Narayan B Miyashita K Hosakawa M. Physiological effects of eicosapentaenoic acid (EPA) and docosahexaenoic acid (DHA) - a review. *Food Rev Int.* (2006) 22:291–307. 10.1080/87559120600694622

[38] Bugeon J Lefevre F Fauconneau B. Fillet texture and muscle structure in brown trout (*Salmo trutta*) subjected to long-term exercise. *Aquacult Res.* (2003) 34:1287–95. 10.1046/j.1365-2109.2003.00938.x

[39] Johnston IA Li XJ Vieira VLA Nickell D Dingwall A Alderson R Muscle and flesh quality traits in wild and farmed Atlantic salmon. *Aquaculture.* (2006) 256:323–36. 10.1016/j.aquaculture.2006.02.048

[40] Purslow PP. Intramuscular connective tissue and its role in meat quality. *Meat Sci.* (2005) 70:435–47. 10.1016/j.meatsci.2004.06.028 22063743

[41] Wright LI Scanga JA Belk KE Engle TE Tatum JD Person RC Benchmarking value in the pork supply chain: characterization of US pork in the retail marketplace. *Meat Sci.* (2005) 71:451–63. 10.1016/j.meatsci.2005.04.024 22060920

[42] Han MY Wang P Xu XL Zhou GH. Low-field NMR study of heat-induced gelation of pork myofibrillar proteins and its relationship with microstructural characteristics. *Food Res Int.* (2014) 62:1175–82. 10.1016/j.foodres.2014.05.062

[43] Li CB Liu DY Zhou GH Xu XL Qi J Shi PP Meat quality and cooking attributes of thawed pork with different low field NMR T21. *Meat Sci.* (2012) 92:79–83. 10.1016/j.meatsci.2011.11.015 22613078

[44] Kumar V Makkar HPS Amselgruber W Amselgruber W Becker K. Physiological, haematological and histopathological responses in common carp (*Cyprinus carpio* L.) fingerlings fed with differently detoxified *Jatropha curcas* kernel meal. *Food Chem Toxicol.* (2010) 48:2063–72. 10.1016/j.fct.2010.05.007 20457206

[45] Cao CH. *A Study on the Difference of Digestive Enzymes and Digestive Tissues Between ice Fish Feeding and Formula Feeding in Snakehead.* Master’s Thesis. Hangzhou: Zhejiang University (2014).

[46] Xie RT Amenyogbe E Chen G Huang JS. Effects of feed fat level on growth performance, body composition and serum biochemical indices of hybrid grouper (*Epinephelus fuscoguttatus* × *Epinephelus polyphekadion*). *Aquaculture.* (2021) 530:735813. 10.1016/j.aquaculture.2020.735813

[47] Cao JX Zhou CY Wang Y Sun YY Pan DD. The effect of oxidation on the structure of G-actin and its binding ability with aroma compounds in carp grass skeletal muscle. *Food Chem.* (2017) 240:346–53. 10.1016/j.foodchem.2017.07.068 28946282

[48] Lefevre F Cos I Pottinger TG Bugeon J. Selection for stress responsiveness and slaughter stress affect flesh quality in pan-size rainbow trout, *Oncorhynchus mykiss*. *Aquaculture.* (2016) 464:654–64. 10.1016/j.aquaculture.2016.07.039

[49] Yao W Wu XY Gao YJ Wu MJ Lu SD Li XJ Effects of replacing fishmeal protein by hemoglobin powder protein on growth performance, food intake, feeding-related gene expression and gut histology of hybrid grouper (*Epinephelus fuscoguttatus* × *Epinephelus lanceolatus*) juveniles. *Aquaculture.* (2018) 488:235–43. 10.1016/j.aquaculture.2018.01.038

[50] Li Y Shi JH Shi SC Liu ZJ Li Z Li JZ. Effect of live, frozen and artificial feeds on digestive enzymes, aminotransferase, histology of liver and intestine in mandarin fish hybrid (*Siniperca chuatsi*♀ × *Siniperca scherzeri*♂). *Israeli J Aquac Bamidgeh.* (2015) 1185:1–8. 10.46989/001c.20701

