# Supplementary material for: The comparison of largemouth bass (Micropterus salmoides) fed trash fish and formula feeds: Growth, flesh quality and metabolomics
Source: Front Nutr. 2022 Sep 30;9:966248. doi: 10.3389/fnut.2022.966248 (PMC9561894; doi:10.3389/fnut.2022.966248)
Supplement: Supplementary file 1 [file Table_1.DOCX]

**Table S1.** Differentially abundant metabolites in self-made formula (SF) group and trash fish (TF) group.

| **Metabolites** | **VIP** | ***P*-Value** | **Fold changes** | **Class** |
| --- | --- | --- | --- | --- |
| L-arabinitol | 1.614208725 | 1.56681E-13 | 8.030395518 | Organooxygen compounds |
| Xylitol | 1.113469747 | 3.34106E-10 | 8.743065539 | Organooxygen compounds |
| (+)-.gamma.-tocopherol | 1.820790118 | 8.97484E-10 | 0.019636208 | Prenol lipids |
| L-glutathione, reduced | 1.802032829 | 1.08142E-09 | 0.041574668 | Carboxylic acids and derivatives |
| 1-o-(9z-octadecenyl)-sn-glycero-2,3-cyclic-phosphate | 1.797300428 | 1.2423E-09 | 0.044323518 |  |
| 1-naphthol-5-sulfonic acid | 1.782614693 | 1.56648E-09 | 0.058489612 | Naphthalenes |
| 1,2-diarachidonoyl-sn-glycero-3-phosphocholine | 5.814490009 | 4.43392E-09 | 2.114381026 | Glycerophospholipids |
| Xanthurenic acid | 2.147836587 | 8.21006E-09 | 119.2257178 | Quinolines and derivatives |
| Asn-Ile-Lys | 1.622669382 | 9.8895E-09 | 0.149644833 | Carboxylic acids and derivatives |
| 1,2-didocosahexaenoyl-sn-glycero-3-phosphocholine | 6.879808845 | 4.19249E-08 | 2.871872283 | Glycerophospholipids |
| 1,2-dioleoyl-sn-glycero-3-phosphatidylcholine | 3.993348412 | 8.30182E-08 | 2.613381855 |  |
| Orphenadrine | 1.577637322 | 1.17213E-07 | 15.55068162 | Benzene and substituted derivatives |
| Guanine | 1.160208952 | 1.35632E-07 | 24.39710292 | Imidazopyrimidines |
| D-myo-inositol-3,4,5,6-tetraphosphate | 1.298249322 | 2.31935E-07 | 0.008692073 | Organooxygen compounds |
| Cetaben | 1.216679939 | 2.89856E-07 | 30.87629556 | Benzene and substituted derivatives |
| 4-hydroxyquinoline | 3.60040643 | 3.79507E-07 | 24.59991483 | Quinolines and derivatives |
| .beta.-carboline-1-propionic acid | 8.406405134 | 5.88258E-07 | 0.045916985 |  |
| Stachydrine | 3.998395067 | 1.33361E-06 | 3.132455567 | Carboxylic acids and derivatives |
| Betaine | 25.41185989 | 1.58457E-06 | 2.120902515 | Carboxylic acids and derivatives |
| Dimethyl sulfoxide | 1.567796594 | 2.03672E-06 | 30.93835938 |  |
| Ephedrine | 8.123252913 | 2.1631E-06 | 60.06004388 | Benzene and substituted derivatives |
| Acenapthylene | 1.405584904 | 3.09349E-06 | 0.2024936 |  |
| N-acetyl-d-glucosamine | 10.52939685 | 3.17234E-06 | 31.58339263 | Organooxygen compounds |
| Aniline | 8.283666034 | 6.20779E-06 | 78.43409121 | Benzene and substituted derivatives |
| 20-hydroxyarachidonic acid | 1.116223387 | 8.04324E-06 | 0.22294691 | Fatty Acyls |
| Thiamine | 2.113485039 | 1.45924E-05 | 47.63776703 | Diazines |
| Glutathione, oxidized | 1.509727483 | 1.78972E-05 | 0.318900857 | Carboxylic acids and derivatives |
| 1-Stearoyl-sn-glycerol 3-phosphocholine | 7.404522928 | 2.11041E-05 | 3.326589824 |  |
| N-methyl-n-(tetrahydro-2-furanylmethyl)-4-piperidinamine | 16.43568338 | 2.59504E-05 | 0.005001054 | Piperidines |
| Ametryne | 2.754579472 | 2.77676E-05 | 843.371121 | Triazines |
| 1,2-di(4z,7z,10z,13z,16z,19z-docosahexaenoyl)-sn-glycero-3-phosphoethanolamine | 1.2117235 | 2.8421E-05 | 1.801478435 | Glycerophospholipids |
| Acetylnorfentanyl | 1.023107718 | 3.35483E-05 | 2.505236941 | Benzene and substituted derivatives |
| Nor-nitrogen mustard | 1.364615174 | 3.55556E-05 | 0.034204353 | Organonitrogen compounds |
| Glutathione | 2.383480018 | 3.5776E-05 | 3.594589837 | Carboxylic acids and derivatives |
| Tosyl-l-lysyl-chloromethane | 1.476652497 | 4.10375E-05 | 0.548320082 | Benzene and substituted derivatives |
| N-nervonoyl-d-erythro-sphingosylphosphorylcholine | 3.723745221 | 6.15199E-05 | 1.570612887 | Sphingolipids |
| Promazine | 1.856094406 | 8.36214E-05 | 0.396619309 | Benzothiazines |
| PC(16:0/16:0) | 4.625311543 | 8.88483E-05 | 0.548377349 | Glycerophospholipids |
| Dehydroascorbic acid | 1.247185376 | 9.27316E-05 | 3.99841604 | Lactones |
| Pro-leu | 7.409177007 | 0.000101316 | 0.761791704 | Carboxylic acids and derivatives |
| Fructoselysine | 1.009153636 | 0.000107212 | 39.36938738 | Carboxylic acids and derivatives |
| 1,5-hexadien-3-ol | 4.798129118 | 0.000114864 | 0.573114962 | Organooxygen compounds |
| Leucine | 4.107225802 | 0.000116635 | 0.614594913 | Carboxylic acids and derivatives |
| Thymine | 8.078517185 | 0.000118656 | 0.043678674 | Diazines |
| N.epsilon.-acetyl-l-lysine | 2.261788011 | 0.000134392 | 0.116363518 | Carboxylic acids and derivatives |
| 2,4-dichlorophenol | 23.7215996 | 0.000142201 | 2.67853781 | Benzene and substituted derivatives |
| Norharmane | 2.716505297 | 0.000147846 | 68.48950061 | Indoles and derivatives |
| 1-palmitoyl-2-docosahexaenoyl-sn-glycero-3-phosphocholine | 9.283508007 | 0.00015389 | 1.585646339 | Glycerophospholipids |
| Thiamine monophosphate | 1.233974442 | 0.000166328 | 4.615077688 | Diazines |
| Heptadecanoic acid | 1.561880232 | 0.000174016 | 0.40151946 | Fatty Acyls |
| 1,2-diamino-2-methylpropane | 2.194128634 | 0.000178123 | 0.551186133 | Organonitrogen compounds |
| Thr-His | 1.734690132 | 0.000187093 | 0.361218224 | Carboxylic acids and derivatives |
| Hypotaurine | 1.534157016 | 0.000198667 | 4.466751455 | Sulfinic acids and derivatives |
| L-homoserine | 1.352577399 | 0.000268916 | 10.95622368 | Carboxylic acids and derivatives |
| Ser-Pro | 1.074960098 | 0.000271039 | 0.511173738 | Carboxylic acids and derivatives |
| 1-palmitoyl-2-linoleoyl-sn-glycero-3-phosphocholine | 9.558446625 | 0.000299387 | 1.512870465 | Glycerophospholipids |
| 5-methyl-5,6-dihydrouracil | 1.433531915 | 0.000318146 | 2.443285283 | Diazines |
| Indole | 1.963267588 | 0.000348119 | 0.602159831 | Indoles and derivatives |
| Histidine | 13.82957512 | 0.000366013 | 0.55853522 | Carboxylic acids and derivatives |
| Doxorubicin | 1.063855324 | 0.000439537 | 0.595653824 |  |
| Histamine | 1.318934824 | 0.000450195 | 0.488061772 | Organonitrogen compounds |
| N-.alpha.-acetyl-l-ornithine | 1.454444499 | 0.000472025 | 0.267433853 | Carboxylic acids and derivatives |
| Myo-inositol | 1.054749539 | 0.000484755 | 1.625633599 | Organooxygen compounds |
| N-3-hydroxyoctanoyl-l-homoserine lactone | 1.041721546 | 0.000514696 | 197.4720955 | Carboxylic acids and derivatives |
| Cynarin | 1.078810026 | 0.000573674 | 6.358363557 | Organooxygen compounds |
| Ng,ng-dimethyl-l-arginine | 15.44785599 | 0.000594304 | 0.568956638 | Carboxylic acids and derivatives |
| Chlorohydroquinone | 1.508634054 | 0.00062446 | 7.358538856 | Phenols |
| (+)-.alpha.-tocopherol | 1.962473617 | 0.000668383 | 0.468170088 | Prenol lipids |
| Lpc 18:2 | 5.710988472 | 0.000751757 | 2.774103068 | Glycerophospholipids |
| Phenol | 1.725563663 | 0.000795507 | 0.498213954 | Phenols |
| Ethephon | 3.269187772 | 0.000808871 | 2.400794767 | Organic phosphonic acids and derivatives |
| 1-stearoyl-2-docosahexaenoyl-sn-glycerol | 2.138901811 | 0.000824734 | 1.377080587 | Glycerolipids |
| Cis-4,7,10,13,16,19-docosahexaenoic acid | 6.143119759 | 0.000828317 | 2.047675874 | Fatty Acyls |
| 4-ketopimelic acid | 3.340383978 | 0.000837707 | 0.207786218 | Keto acids and derivatives |
| Pyroglu-Ala-Arg | 1.114372456 | 0.000896348 | 1.497823556 | Carboxylic acids and derivatives |
| Erucamide | 3.271096449 | 0.000930889 | 1.50431231 | Fatty Acyls |
| D-ornithine | 6.750223057 | 0.000944087 | 0.117881699 | Carboxylic acids and derivatives |
| Ornithine | 7.661554218 | 0.001065597 | 0.167412641 | Carboxylic acids and derivatives |
| Tetraethylene glycol | 1.517907294 | 0.001073923 | 1.438715132 | Organooxygen compounds |
| 4-aminobenzoate | 2.49330651 | 0.001095514 | 2.152222573 | Benzene and substituted derivatives |
| L-ng-monomethylarginine | 1.826583736 | 0.001116433 | 0.209604932 | Carboxylic acids and derivatives |
| 3-aminopyrazine-2-carboxylic acid | 1.383565509 | 0.00114998 | 0.589940133 | Diazines |
| Val-Pro | 1.047058465 | 0.001182794 | 0.685299308 | Carboxylic acids and derivatives |
| 1-oleoyl-2-myristoyl-sn-glycero-3-phosphocholine | 3.51894923 | 0.001197209 | 0.639302797 | Glycerophospholipids |
| Imidazole | 1.077957005 | 0.001220891 | 1.633438959 | Azoles |
| Urocanate | 1.103390574 | 0.001368505 | 0.506262473 | Azoles |
| Coniferyl alcohol | 1.718256759 | 0.001396191 | 0.230386129 | Phenols |
| N6,n6-dimethyllysine | 7.086663985 | 0.001524649 | 0.550529261 | Carboxylic acids and derivatives |
| Oleoyl ethylamide | 1.214484914 | 0.00156097 | 5.236913358 | Fatty Acyls |
| Cys-Lys | 1.478453167 | 0.001647606 | 0.012278358 | Carboxylic acids and derivatives |
| Linoleic acid | 5.014918712 | 0.001687175 | 1.770055711 | Fatty Acyls |
| 1',3'-bis[1,2-dilinoleoyl-sn-glycero-3-phospho]-sn-glycerol | 1.407722358 | 0.001784381 | 2.941568769 | Glycerophospholipids |
| Trans-cinnamic acid | 1.214966927 | 0.001797067 | 0.243436859 | Cinnamic acids and derivatives |
| O-succinyl-l-homoserine | 1.577452439 | 0.001991253 | 0.034562686 | Carboxylic acids and derivatives |
| Desisopropyldisopyramide | 2.119212633 | 0.00200135 | 1.900176434 | Carboxylic acids and derivatives |
| Glutathione ethyl ester | 1.439541473 | 0.002157716 | 0.048895741 | Carboxylic acids and derivatives |
| N-.alpha.-(tert-butoxycarbonyl)-l-histidine | 8.627323469 | 0.002254863 | 0.574096337 | Carboxylic acids and derivatives |
| Guanidinoethyl sulfonate | 1.096882528 | 0.002281135 | 1.40354109 | Organic sulfonic acids and derivatives |
| DL-arginine | 12.15271466 | 0.002745857 | 0.306104877 | Carboxylic acids and derivatives |
| Homogentisic acid | 1.219518383 | 0.00306686 | 4.604874537 | Benzene and substituted derivatives |
| 2-methylpyrrolidine | 2.7632193 | 0.003192631 | 0.667129353 |  |
| 5s-hydroxy-6e,8z,11z,14z-eicosatetraenoic acid | 3.041814918 | 0.003265281 | 1.943837028 | Fatty Acyls |
| Magnolol | 3.902307611 | 0.00329815 | 0.579484964 | Benzene and substituted derivatives |
| 1-palmitoyl-sn-glycero-3-phosphocholine | 5.010133606 | 0.00338601 | 1.412397587 | Glycerophospholipids |
| Palmitoyl sphingomyelin | 3.096087966 | 0.003809946 | 1.183146303 | Sphingolipids |
| Penicillic acid | 2.658327924 | 0.003843785 | 0.473000892 | Keto acids and derivatives |
| Glycine | 1.305534561 | 0.003938365 | 0.48232082 | Carboxylic acids and derivatives |
| 4-hydroxybenzaldehyde | 1.921133766 | 0.003938559 | 3.553656807 | Organooxygen compounds |
| Indole-3-butyric acid | 1.896950396 | 0.004010939 | 0.689585275 | Indoles and derivatives |
| DL-threonine | 3.316760821 | 0.004095562 | 0.475722127 | Carboxylic acids and derivatives |
| Linolenic acid | 1.920574462 | 0.004154229 | 0.529828074 | Fatty Acyls |
| N.epsilon.-methyl-l-lysine | 1.690089704 | 0.004249716 | 0.55008734 | Carboxylic acids and derivatives |
| 3,3'-dimethoxybenzidine | 1.418745298 | 0.004306018 | 11.25132234 | Benzene and substituted derivatives |
| 4-aminopyridine | 1.665283582 | 0.004453601 | 0.527585764 | Pyridines and derivatives |
| Inosine | 5.728045909 | 0.004540281 | 1.792997359 | Purine nucleosides |
| L-Glutamine | 3.02480741 | 0.004855621 | 0.349617334 | Carboxylic acids and derivatives |
| Ergothioneine | 1.019703947 | 0.004988681 | 0.542248579 | Carboxylic acids and derivatives |
| Phenylacetyl-l-glutamine | 3.458748107 | 0.005029568 | 0.330456638 | Carboxylic acids and derivatives |
| Ectoine | 1.724390456 | 0.005094328 | 3.70242615 | Carboxylic acids and derivatives |
| Pro-hyp | 4.494518017 | 0.005102255 | 0.550027421 | Carboxylic acids and derivatives |
| Dl-homocysteine | 1.188314169 | 0.005339896 | 2.281219075 | Carboxylic acids and derivatives |
| 1-methylhistidine | 4.972597587 | 0.005519304 | 0.335479339 | Carboxylic acids and derivatives |
| Triflumuron | 1.931430165 | 0.005800899 | 1.528343006 | Benzene and substituted derivatives |
| D-glutamine | 2.769572033 | 0.006900617 | 0.451686296 | Carboxylic acids and derivatives |
| 4-piperidinecarboxamide | 1.204766801 | 0.007079665 | 0.347233565 | Piperidines |
| .gamma.-hexalactone | 3.398149261 | 0.00712173 | 0.708638921 | Lactones |
| Alfentanyl | 1.225691664 | 0.007348534 | 10.75060141 | Benzene and substituted derivatives |
| 1-o-hexadecyl-2-o-(4z,7z,10z,13z,16z,19z-docosahexaenoyl)-sn-glyceryl-3-phosphorylcholine | 2.50125396 | 0.007625668 | 0.745703121 | Glycerophospholipids |
| Lysine | 6.427251858 | 0.008324427 | 0.221319858 | Carboxylic acids and derivatives |
| Isophorone | 1.128577135 | 0.008983439 | 1.799230106 | Organooxygen compounds |
| Melphalan | 1.275709525 | 0.009152342 | 2.370917216 | Carboxylic acids and derivatives |
| D-erythro-imidazolylglycerol phosphate | 10.99792147 | 0.009747676 | 0.629815862 | Organic phosphoric acids and derivatives |
| .beta.-hydroxypropionic acid | 1.428494766 | 0.009829597 | 0.538287239 | Hydroxy acids and derivatives |
| 1-Aminocyclopropanecarboxylic acid | 1.52282314 | 0.009905765 | 0.478811558 | Carboxylic acids and derivatives |
| D-threonine | 2.17669918 | 0.009911167 | 0.528792381 | Carboxylic acids and derivatives |
| D-Mannose | 2.244385585 | 0.010017393 | 0.496492282 | Organooxygen compounds |
| L-Histidinol phosphate | 1.173760645 | 0.010263997 | 1.950229929 |  |
| N-lauroyl-d-erythro-sphingosylphosphorylcholine | 1.575212127 | 0.010792553 | 0.609889935 | Sphingolipids |
| N-acetylneuraminic acid, 2,3-dehydro-2-deoxy- | 1.058707964 | 0.01185117 | 1.529166761 | Organooxygen compounds |
| DL-Lysine | 1.378356227 | 0.012967217 | 0.500415147 | Carboxylic acids and derivatives |
| Psoralidin | 1.174594322 | 0.013525105 | 2.409467461 | Isoflavonoids |
| 2-Oxoadipic acid | 1.021796307 | 0.014045853 | 0.757953704 | Keto acids and derivatives |
| Pyruvate | 16.9866092 | 0.014337407 | 0.532811698 | Keto acids and derivatives |
| 4-[5-[[4-[5-[acetyl(hydroxy)amino]pentylamino]-4-oxobutanoyl]-hydroxyamino]pentylamino]-4-oxobutanoic acid | 1.236188991 | 0.014854071 | 2.444335445 | Fatty Acyls |
| 4-hydroxyhexenal | 3.399468814 | 0.015946698 | 0.574523263 | Organooxygen compounds |
| Phosphocholine | 2.68014986 | 0.016060296 | 1.422353084 | Organonitrogen compounds |
| Thioetheramide-PC | 4.578271237 | 0.016421092 | 1.457308007 |  |
| D-ribose 5-phosphate | 1.605074196 | 0.01673049 | 1.635606552 | Organooxygen compounds |
| Oxypurinol | 1.021339345 | 0.016989094 | 2.363141949 | Imidazopyrimidines |
| Hypoxanthine | 3.873239271 | 0.017245492 | 1.801489079 | Imidazopyrimidines |
| 4-methyl-1h-pyrazole | 2.146953464 | 0.017888014 | 0.612587749 | Azoles |
| Chenodeoxycholate | 1.46131298 | 0.017903167 | 0.816969403 | Steroids and steroid derivatives |
| 3-pyridinemethanol | 2.016977373 | 0.01837334 | 0.580568955 |  |
| Trimethylamine n-oxide | 7.070415598 | 0.018472679 | 1.167918473 | Organonitrogen compounds |
| DL-proline | 9.010256132 | 0.018979039 | 0.625858515 | Carboxylic acids and derivatives |
| (r)-(+)-arachidonyl-1'-hydroxy-2'-propylamide | 1.790563009 | 0.019565473 | 1.777074485 | Fatty Acyls |
| Succinate | 2.507034268 | 0.020271546 | 1.228111597 | Carboxylic acids and derivatives |
| 1-palmitoyl-2-hydroxy-sn-glycero-3-phosphoethanolamine | 1.209180006 | 0.020443503 | 1.880174692 | Glycerophospholipids |
| Isovaleryl-l-carnitine | 3.245208313 | 0.020630452 | 2.082832046 | Fatty Acyls |
| Cholesterol | 2.231607074 | 0.020978587 | 1.64921796 | Steroids and steroid derivatives |
| Methylphosphonic acid | 1.103206656 | 0.021314727 | 1.361583787 | Organic phosphonic acids and derivatives |
| Fenfluramine | 3.026426564 | 0.021978971 | 1.863951409 | Benzene and substituted derivatives |
| Niacinamide | 24.16560543 | 0.022645356 | 1.970343581 | Pyridines and derivatives |
| Phenylalanine | 2.187216935 | 0.022860716 | 1.275660695 | Carboxylic acids and derivatives |
| D-fructose | 1.08389711 | 0.024629161 | 1.763774183 | Organooxygen compounds |
| 14-hydroxy-4z,7z,10z,12e,16z,19z-docosahexaenoic acid | 1.298058908 | 0.024979811 | 0.064140169 | Fatty Acyls |
| Latanoprost | 3.868490371 | 0.026555277 | 15.18041889 | Fatty Acyls |
| D-myo-inositol-1,4-diphosphate | 2.183054745 | 0.027033107 | 1.64047323 | Organooxygen compounds |
| Ethoxyquin | 1.053230635 | 0.031264287 | 12.00827653 | Quinolines and derivatives |
| Cis-9-palmitoleic acid | 1.824871773 | 0.032606926 | 0.633177761 | Fatty Acyls |
| Uracil | 2.406552274 | 0.033052548 | 1.972464874 | Diazines |
| .beta.-d-glucose | 1.230730949 | 0.034283223 | 1.537454598 | Organooxygen compounds |
| 1-o-hexadecyl-2-o-acetyl-sn-glyceryl-3-phosphorylcholine | 3.128938704 | 0.037635749 | 1.28268586 | Glycerophospholipids |
| Triethanolamine | 1.639908001 | 0.041801023 | 1.493956176 | Organonitrogen compounds |
| Alpha-d-glucose 1,6-bisphosphate | 1.037503296 | 0.045333418 | 2.823144998 | Organooxygen compounds |
| Dl-malic acid | 7.116659416 | 0.045600876 | 1.456217559 | Hydroxy acids and derivatives |
| Pyrocatechol | 2.446293403 | 0.049171151 | 0.644814634 | Phenols |

^1^The metabolite mean intensities are presented as fold changes compared to flesh tissue of TF group.
